# Hypoxia‐responsive *ERFs* involved in postdeastringency softening of persimmon fruit

**DOI:** 10.1111/pbi.12725

**Published:** 2017-04-11

**Authors:** Miao‐miao Wang, Qing‐gang Zhu, Chu‐li Deng, Zheng‐rong Luo, Ning‐jing Sun, Donald Grierson, Xue‐ren Yin, Kun‐song Chen

**Affiliations:** ^1^ Zhejiang Provincial Key Laboratory of Horticultural Plant Integrative Biology Zhejiang University Hangzhou China; ^2^ The State Agriculture Ministry Laboratory of Horticultural Plant Growth Development and Quality Improvement Zhejiang University Hangzhou China; ^3^ Key Laboratory of Horticultural Plant Biology Ministry of Education Huazhong Agricultural University Wuhan China; ^4^ Department of Horticultural Sciences College of Agriculture Guangxi University Nanning China; ^5^ Plant & Crop Sciences Division School of Biosciences University of Nottingham Loughborough UK

**Keywords:** Astringency removal, *ERF*, high CO_2_, hypoxia, persimmon fruit, postharvest softening, transcriptional regulation

## Abstract

Removal of astringency by endogenously formed acetaldehyde, achieved by postharvest anaerobic treatment, is of critical importance for many types of persimmon fruit. Although an anaerobic environment accelerates de‐astringency, it also has the deleterious effect of promoting excessive softening, reducing shelf life and marketability. Some hypoxia‐responsive ethylene response factors (*ERFs*) participate in anaerobic de‐astringency, but their role in accelerated softening was unclear. Undesirable rapid softening induced by high CO_2_ (95%) was ameliorated by adding the ethylene inhibitor 1‐MCP (1 μL/L), resulting in reduced astringency while maintaining firmness, suggesting that CO_2_‐induced softening involves ethylene signalling. Among the hypoxia‐responsive genes, expression of eight involved in fruit cell wall metabolism (*Dk*β*‐gal1/4*,* DkEGase1*,* DkPE1/2*,* DkPG1*,* DkXTH9*/10) and three ethylene response factor genes (*DkERF8/16/19*) showed significant correlations with postdeastringency fruit softening. Dual‐luciferase assay indicated that *DkERF8/16/19* could trans‐activate the *DkXTH9* promoter and this interaction was abolished by a mutation introduced into the C‐repeat/dehydration‐responsive element of the *DkXTH9* promoter, supporting the conclusion that these DkERFs bind directly to the *DkXTH9* promoter and regulate this gene, which encodes an important cell wall metabolism enzyme. Some hypoxia‐responsive *ERF* genes are involved in deastringency and softening, and this linkage was uncoupled by 1‐MCP. Fruit of the Japanese cultivar ‘Tonewase’ provide a model for altered anaerobic response, as they lost astringency yet maintained firmness after CO_2_ treatment without 1‐MCP and changes in cell wall enzymes and ERFs did not occur.

## Introduction

Plant responses to anoxia involve a range of metabolic and morphological changes on different timescales, including rapid induction of anaerobic metabolism (Kennedy *et al*., 1992; Voesenek and Bailey‐Serres, 2015). For persimmon (*Diospyros kaki*), the anaerobic metabolite acetaldehyde, which accumulates under high CO_2_ treatment (95%), participates in fruit postharvest deastringency by converting the soluble tannins to insoluble products (Min *et al*., 2012; Pesis and Ben‐Arie, 1984; Taira *et al*., 1992, 2001). However, deastringency is usually also accompanied by rapid fruit softening (Arnal and Del Río, 2004; Yin *et al*., 2012). Thus, although useful for taste improvement, anaerobic treatment has very adverse effects on persimmon fruit storage life.

Softening in most fruit occurs naturally at the commencement of ripening and continues after harvest. It is due primarily to partial cell wall degradation and a reduction in intercellular adhesion (Li *et al*., 2010) catalysed by a battery of enzymes including pectin methylesterase (PME, EC 3.1.1.11), polygalacturonase (PG, endo‐type, EC 3.2.1.15; exo‐type, EC 3.2.1.67) and β‐galactosidase (β‐gal, EC 3.2.1.23) (Brummell and Harpster, 2001; Payasi *et al*., 2009; Vicente *et al*., 2007). Understanding the roles of individual genes and enzymes in changing fruit texture has continued to advance since the first experiments on *PG* antisense transgenic tomato (Smith *et al*., 1988). Some reports indicated that modulation of individual genes effectively influenced fruit texture/softening, such as *PG* (Carrington *et al*., 1993; Kramer *et al*., 1992), *TBG4* (a β‐gal gene, Smith *et al*., 2002), *Pmeu1* (a *pectinesterase* gene, Phan *et al*., 2007) and *PL* (pectate lyase, Silvia *et al*., 2002). Additional research has indicated that multiple genes contribute to fruit texture, and in tomato, *Fir* ^*s.p*.^ QTL2.5 (containing three *PME* genes) is tightly correlated with fruit firmness (Chapman *et al*., 2012) and double‐suppression of *LePG* and *LeEXP1* resulted in increased firmness compared to single gene repression (Powell *et al*., 2003). Recently, a tomato pectate lyase has been implicated in playing a major role in reducing cell adhesion and firmness (Uluisik *et al*., 2016). Many of these investigations have been conducted in tomato and strawberry. Limited studies on cell wall‐related genes in persimmon have highlighted the importance for fruit softening, of genes such as *DkXTH1* and *DkXTH2*, encoding xyloglucan endotransglycosylase/hydrolases (Nakatsuka *et al*., 2011; Zhu *et al*., 2013) and *DkExp3*, which encodes an expansin (Zhang *et al*., 2012).

Plants respond to hypoxia by the N‐end rule pathway which post‐translationally regulates levels of group VII ERFs (Gibbs *et al*., 2011; Licausi *et al*., 2011). Among other responses, this induces alcohol dehydrogenase (*ADH*) and pyruvate decarboxylase (*PDC*) genes (Hinz *et al*., 2010; Licausi *et al*., 2010; Papdi *et al*., 2015; Yang *et al*., 2011) which generate the acetaldehyde which in persimmon fruit precipitates soluble tannins. Several transcription factors have been implicated in regulating this process, including ethylene response factors (*ERFs*), and some may also be involved in the softening that accompanies deastringency. Some AP2/ERF transcription factors have also been associated with fruit cell degradation and softening in various other fruit (Xie *et al*., 2016), such as kiwifruit *AdERF9*, which is a transcriptional repressor acting on the *AdXET5* promoter (Yin *et al*., 2010), and *MdCBF* (an AP2/ERF member), which activates *MdPG1* (Tacken *et al*., 2010). Using introgression lines, *SlERF2.2* was shown to underlie the firmness QTL, *Fir* ^*s.p*.^ QTL2.2, and its expression is tightly correlated with fruit texture (Chapman *et al*., 2012). Another *AP2/ERF* gene, *SlAP2a*, is also associated with retarding fruit softening, as *SlAP2a* antisense tomatoes are softer than wild‐type fruit (Chung *et al*., 2010), and *LeERF1* antisense transgenic fruit had significant longer shelf life (Li *et al*., 2007). However, the hypoxia‐responsive ERFs have mainly been investigated for their regulation of anaerobic related genes, and their possible relationship with other target genes is largely unknown.

Twenty‐two hypoxia‐responsive *DkERF* genes have been isolated from persimmon (Min *et al*., 2012, 2014; Yin *et al*., 2012) and *DkERF9/10/19/22* were shown to be involved directly in transcriptional regulation of anaerobic metabolism genes involved in persimmon deastringency (Min *et al*., 2012, 2014), but a possible role in postdeastringency fruit softening was not investigated. In the present research, a combination of high CO_2_ storage and 1‐MCP treatment (together with CO_2_ treatment) was shown to maintain fruit firmness while removing astringency, implicating some ERFs in the excessive postdeastringency softening. The roles of *DkERFs* in controlling cell wall metabolism‐related genes were analysed during these treatments and in a Japanese cultivar ‘Tonewase’, which appears to have an altered anaerobic response, and lacks the ethylene and softening response to high CO_2_.

## Results

### Effects of CO_2_ and 1‐MCP on ‘Mopanshi’ persimmon deastringency and softening

**Figure 1:**
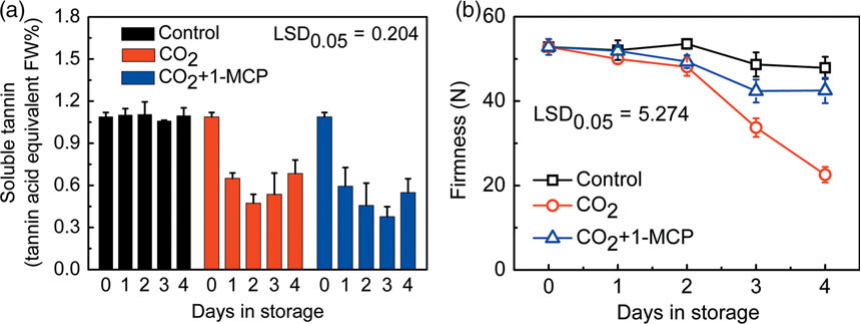
Effects of CO_2_ and CO_2_ + 1‐MCP treatments on soluble tannins (a) and firmness (b) in ‘Mopanshi’ persimmon fruit at 20 °C. The persimmon fruit were treated with CO_2_ (95%) and CO_2_ + 1‐MCP (95% CO_2_ and 1 μL/L 1‐MCP) for 1 day, while control fruit were sealed in airtight containers. All treatments and subsequent storage was at 20 °C. Error bars indicate SEs from 3 (for soluble tannins) or 10 (for firmness) replicates.

Mature ‘Mopanshi’ persimmon fruit were astringent at harvest and the soluble tannin content was maintained at approximately 1.1%, during 4‐day storage (Figure 1a). CO_2_ treatment (95%, 1 day) caused a decline in soluble tannins to 0.65% after 1 day and 0.47% after 2 day (Figure 1a). This was accompanied by a rapid decrease in firmness in CO_2_‐treated fruit, to 33.7 N at 3 day and 22.6 N at 4 day, compared to control fruit firmness of 48.7 N at 3 day and 47.9 N at 4 day (Figure 1b).

The effects of adding the ethylene action antagonist 1‐MCP (1 μL/L) to the CO_2_ (95%) treatment were investigated in order to test whether this could alleviate the rapid softening that occurred during astringency removal. The results indicated that 1‐MCP‐treated fruit had higher firmness than the control fruit in CO_2_ alone, with 42.4 N at 3 days and 42.5 N at 4 days, slightly lower than control fruit in air (Figure 1b). CO_2_ + 1‐MCP also enhanced ‘Mopanshi’ persimmon astringency removal, as indicated by the decrease in soluble tannin, to 0.59% after 1 day and 0.46% after 2 day, values which were similar to those in CO_2_‐treated fruit without 1‐MCP (Figure 1a). Similar effects were confirmed in a subsequent replication in a different year with ‘Mopanshi’ fruit (data not shown).

### Isolation and analysis of deastringency‐responsive cell wall degradation‐related and *DkERF* genes associated with ‘Mopanshi’ persimmon softening

Using the previously generated RNA‐seq data (Min *et al*., 2014), deastringency‐responsive cell wall‐related unigenes were obtained. After RACE experiments, 35 genes were cloned encoding the following cell wall degrading enzymes: α‐L‐arabinofuranosidase (*DkAraf1‐2*, KX259530–KX259531), endoglucanase (*DkEGase1‐2*, KX259532–KX259533), β‐galactosidase (*Dk*β*‐gal1‐8*, KX259534–KX259541), Mannan endo‐1,4‐beta‐mannosidase (*DkMAN1*, KX259542), polygalacturonase (*DkPG1*, Jiang *et al*., 2010; *DkPG2‐5*, KX259551–KX259554), pectinesterase (*DkPE1‐8*, KX259543–KX259550), pectate lyase (*DkPL1*, KX259555) and xyloglucan endotransglycosylase/hydrolase (*DkXTH1‐2*,* DkXTH4*, Han *et al*., 2015; *DkXTH8‐9*, KF318888‐9; *DkXTH10‐12*, KX259556‐KX259558).

**Figure 2:**
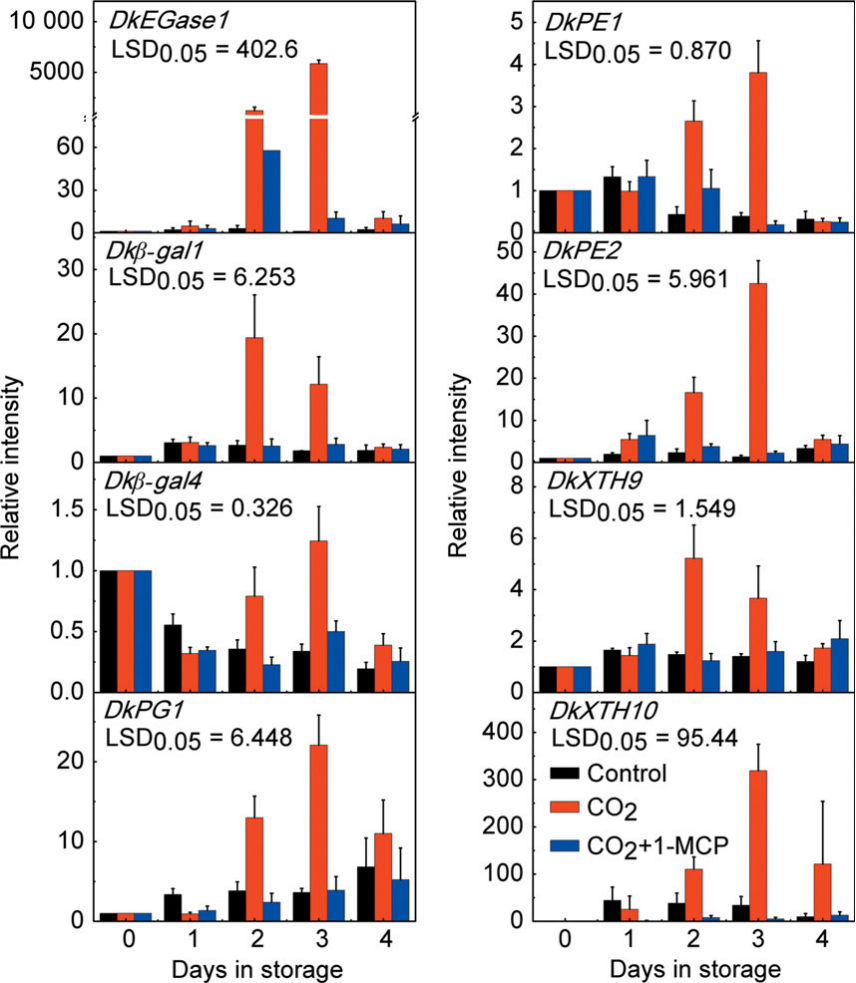
Accumulation of mRNAs from eight cell wall‐related genes in response to CO_2_ and CO_2_ + 1‐MCP treatments in ‘Mopanshi’ persimmon fruit at 20 °C. Gene expression was analysed by real‐time PCR. Error bars indicate SEs from three replications. *DkEGase1*: endoglucanase; *Dk*β*‐gal1/4*: β‐galactosidase; *DkPG1*: polygalacturonase; *DkPE1/2*: pectinesterase; *DkXTH9/10*: xyloglucan endotransglycosylase/hydrolase.

These genes were all expressed in fruit but responded differentially to CO_2_ treatment, with only *DkEGase1*,* Dk*β*‐gal1*,* Dk*β*‐gal4*,* DkPG1*,* DkPG4*,* DkPE1*,* DkPE2*,* DkXTH1*,* DkXTH4*,* DkXTH9‐11* showing a strong increase, while the others were nonresponsive or were repressed by CO_2_ treatment (Figures 2, S1). Among the CO_2_ treatment‐induced genes, *DkEGase1* and *DkXTH10* were highly responsive, and at 2 days, their mRNA abundance increased approximately 1233‐ and 110‐fold, respectively (Figure 2). Furthermore, increases in mRNA from most of the CO_2_‐induced cell wall degrading genes were reduced in CO_2_ + 1‐MCP‐treated fruit, with the exception of *DkPG4*,* DkXTH1*,* DkXTH4* and *DkXTH11*. For instance, *DkXTH1* mRNA was enhanced 6.36‐fold at 1 day by CO_2_ treatment, and a similar increase was found in CO_2_ + 1‐MCP‐treated fruit (5.71‐fold at 1 day; Figure S1). Thus, of the 35 cell wall degradation‐related genes, *DkEGase1*,* Dk*β*‐gal1*,* Dk*β*‐gal4*,* DkPG1*,* DkPE1*,* DkPE2*,* DkXTH9* and *DkXTH10* were the most likely to be involved in major persimmon fruit softening during and after CO_2_ treatment (Figures 2 and S1).

**Figure 3:**
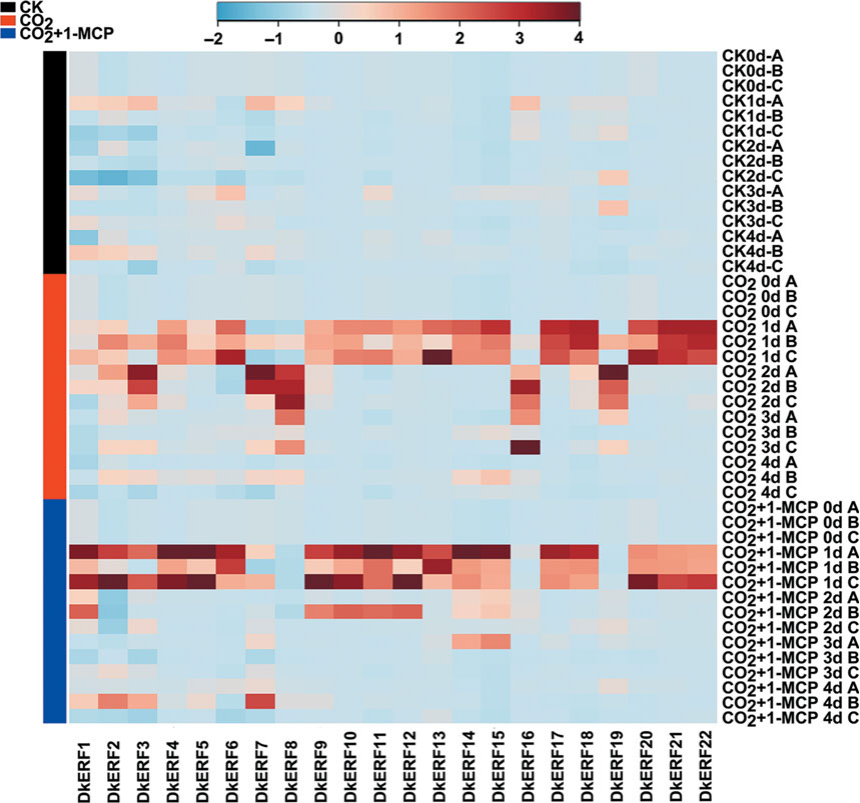
Accumulation of mRNA from hypoxia‐responsive *DkERF* genes in response to CO_2_ and CO_2_ + 1‐MCP treatments in ‘Mopanshi’ persimmon fruit at 20 °C. Gene expression was analysed by real‐time PCR. The heatmap was constructed by MetaboAnalyst 3.0 and indicates the mRNA abundance. A, B and C represent three biological replicates. *DkERF1‐22*: ethylene response factors.

mRNAs from twenty‐two *DkERF* genes were found to increase in abundance during deastringency treatment (high CO_2_); however, only four, *DkERF9/10/19/22*, have been shown previously to be involved in transcriptional regulation of anoxia‐related genes during persimmon fruit deastringency (Min *et al*., 2012, 2014). The possibility that other *DkERF* genes might also participate in fruit softening during astringency removal was investigated. Using the ‘Mopanshi’ persimmon, expression of twenty‐two *DkERF* genes was analysed and mRNAs for all of these were up‐regulated by CO_2_ treatment (Figure 3). Adding 1‐MCP blocked the enrichment of four *DkERF* genes transcripts, *DkERF7*,* DkERF8*,* DkERF16* and *DkERF19*, suggesting they could play a role in fruit softening (Figures 1b, 3). One, *DkERF7*, contained an EAR motif within the coding region and is a putative transcriptional repressor (Kagale and Rozwadowski, 2011), while the accumulation of mRNAs from the other three *DkERF* genes was positively correlated with fruit softening.

### Effect of CO_2_ and CO_2_ + 1‐MCP treatments on persimmon fruit deastringency and softening in various cultivars

**Figure 4:**
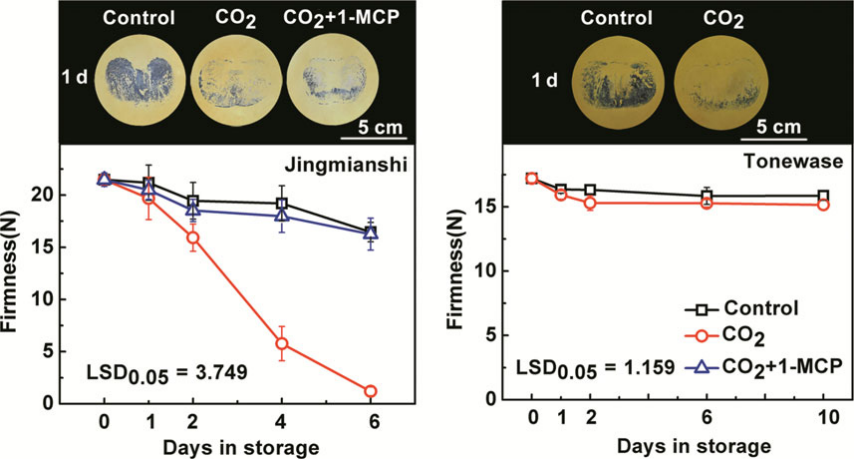
Changes in soluble tannins and firmness in two astringent cultivars, ‘Jingmianshi’ and ‘Tonewase’, in response to postharvest treatments. The Chinese cultivar ‘Jinmianshi’ fruit were treated with CO_2_ (95%) or CO_2_ + 1‐MCP (95% CO_2_ and 1 μL/L 1‐MCP) for 1 day, separately. Japanese astringent cultivar ‘Tonewase’ fruit were only treated with CO_2_ (95%) for 1 day. Control fruit was sealed in airtight containers. All treatments and subsequent storage were at 20 °C. Astringency was indicated by soluble tannin content, using the tannin printing method. The black colour indicates soluble tannins and the intensity of black reflects the soluble tannin content. Error bars indicate SEs from eight replicates.

In order to confirm the effect of CO_2_ + 1‐MCP treatment on persimmon fruit deastringency and softening, another Chinese astringent‐type cultivar, ‘Jingmianshi’, was studied. CO_2_ (95%, 1 day) treatment effectively accelerated deastringency as indicated by the darker staining from tannin printing of control fruit, compared with treated fruit (Figure 4). CO_2_ + 1‐MCP treatment had similar effects on deastringency as CO_2_ alone (Figure 4), but there were major differences in fruit softening (Figure 4). Whereas the firmness of ‘Jingmianshi’ fruit in CO_2_ decreased from 21.48 N at 0 day to 1.19 N at 6 day, fruit in CO_2_ + 1‐MCP retained a firmness of 16.26 N at 6 day (Figure 4).

A comparison was made with a Japanese astringent cultivar, ‘Tonewase’, which responded similarly to deastringency in CO_2_ (95%, 1 day) but did not exhibit the rapid softening observed in the Chinese cultivars, even in the absence of 1‐MCP (Figure 4). During the 10‐day storage, both CO_2_‐treated and control fruit firmness decreased only slightly from 17.2 N at 0 day to 15.86 N and 15.15 N at 10 days for control and CO_2_‐treated fruit, respectively.

### Accumulation of mRNAs from fruit softening‐related genes in various cultivars

**Figure 5:**
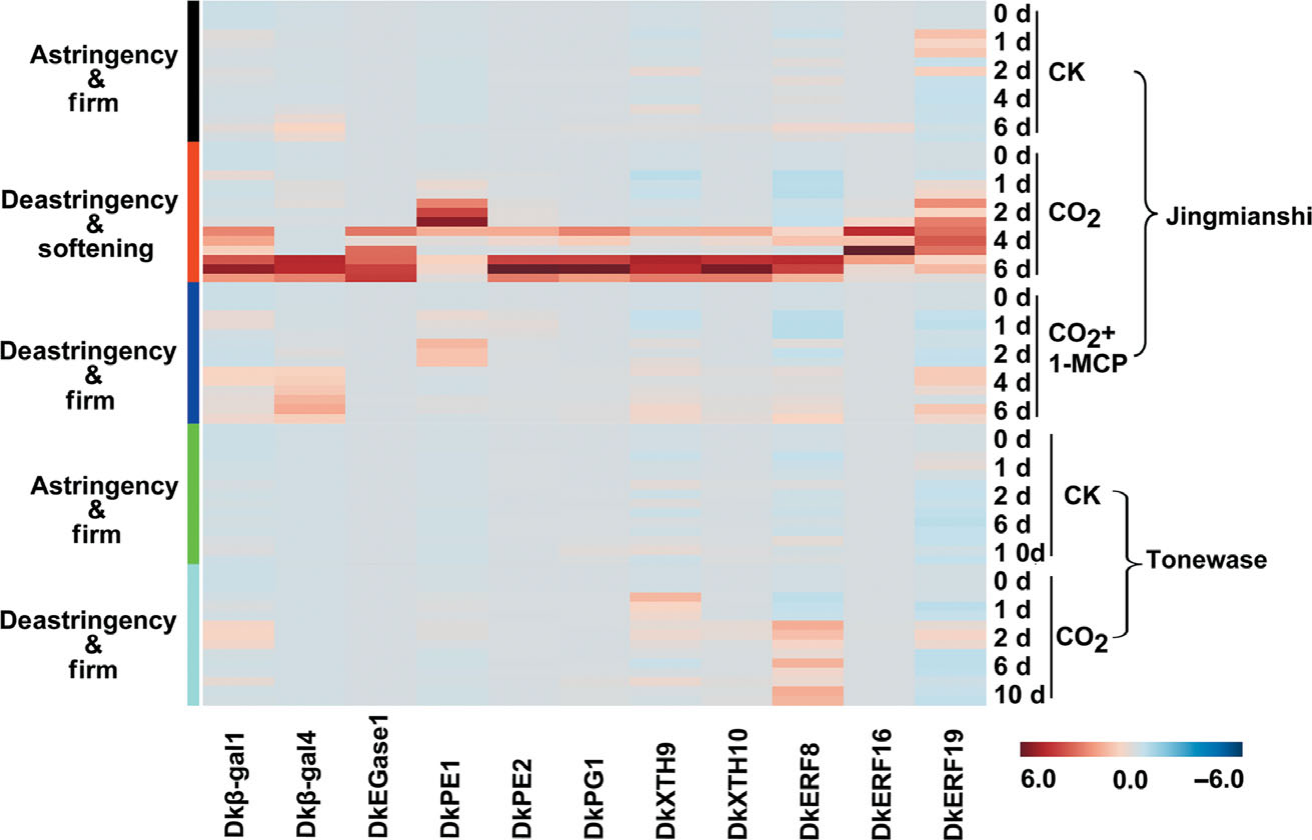
Relationship between accumulation of mRNAs from cell wall‐related genes, *DkERF* and persimmon fruit softening in different cultivars. The astringent Chinese cultivar ‘Jingmianshi’ was treated with CO_2_ and CO_2_ + 1‐MCP, while the Japanese astringent cultivar ‘Tonewase’ was only treated with CO_2_. The concentration of CO_2_ and 1‐MCP was 95% and 1 μL/L. All treatments and post‐treatment storage were conducted at 20 °C. Gene expression was analysed by real‐time PCR. The heatmap was constructed by MetaboAnalyst 3.0 and indicates the mRNA abundance. *DkEGase1*: endoglucanase; *Dk*β*‐gal1/4*: β‐galactosidase; *DkPG1*: polygalacturonase; *DkPE1/2*: pectinesterase; *DkXTH9/10*: xyloglucan endotransglycosylase/hydrolase; *DkERF8/16/19*: ethylene response factors.

Using ‘Jingmianshi’ and ‘Tonewase’, the involvement of the selected eight cell wall‐related genes and three *DkERF* genes in fruit softening was assessed. The heatmap (Figure 5) shows that these eleven genes were all highly expressed in CO_2_‐treated fruit of the ‘Jingmianshi’ cultivar, which undergoes both deastringency and rapid softening, and were significantly inhibited in the CO_2_ + 1‐MCP treatment, where fruit underwent deastringency but remained firm. In the astringent Japanese cultivar ‘Tonewase’, however, mRNAs for these eleven genes remained at basal levels in response to CO_2_ treatment alone, resulting in deastringency but maintaining firmness (Figure 5). Taking the results from the three cultivars ‘Mopanshi’, ‘Jingmianshi’ and ‘Tonewase’ together, accumulation of mRNAs for these eleven genes, encoding five cell wall enzymes (*DkEGase1*,* Dk*β*‐gal1*,* Dk*β*‐gal4*,* DkPG1*,* DkPE1*,* DkPE2*,* DkXTH9*,* DkXTH10*) and three ERF transcription factors (*DkERF8*,* DkERF16* and *DkERF19*) is highly correlated with CO_2_‐triggered softening associated with deastringency in Chinese cultivars of persimmon.

### Roles of softening‐related *DkERFs* in controlling expression of genes encoding cell wall degrading enzymes

**Figure 6:**
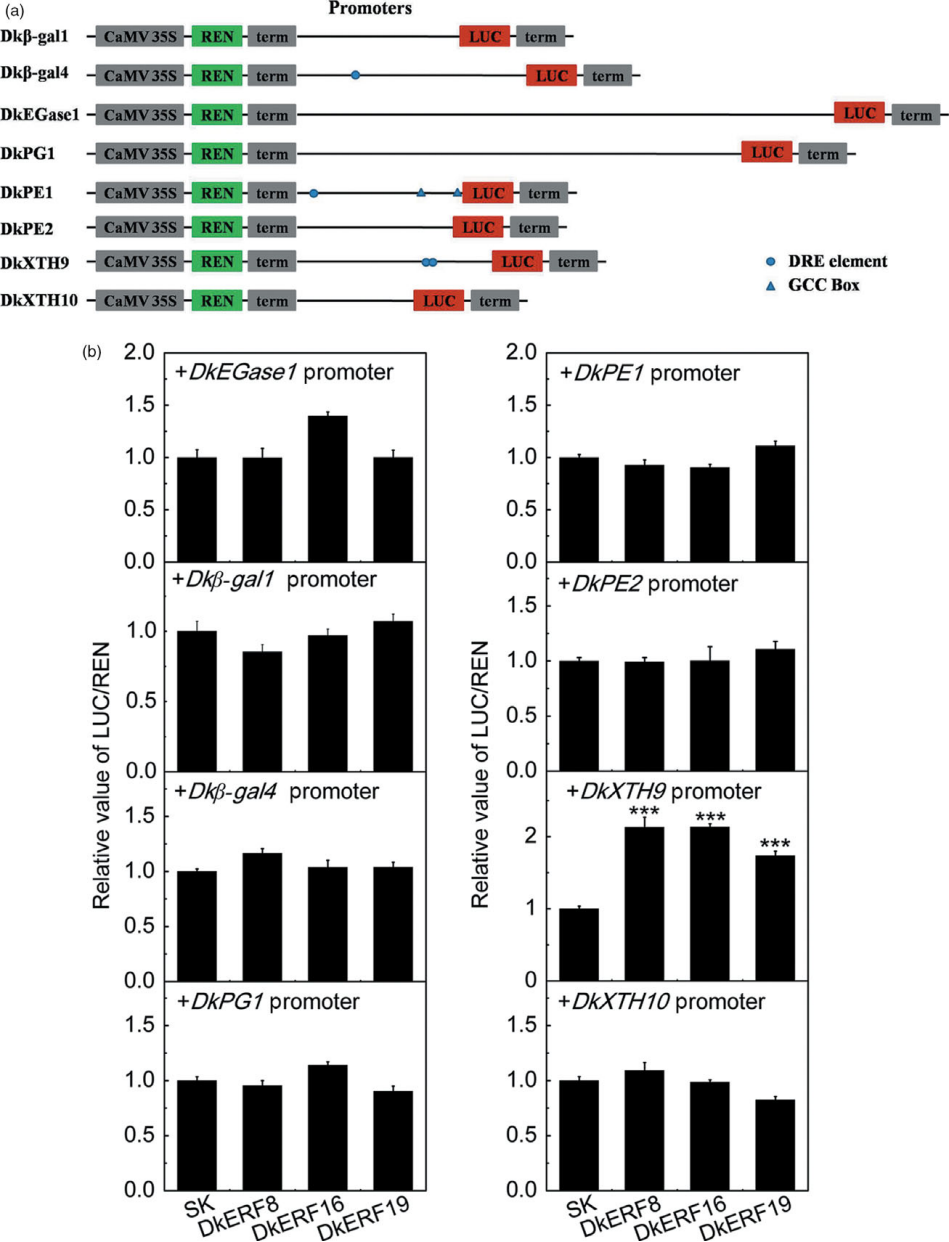
Effect of DkERF on transcription from the promoters of cell wall metabolism‐related genes. (a) Schematics of promoters: lines indicate promoter length, triangles show GCC box, circles represent DRE motifs, and triangles indicate GCC boxes. (b) *In vivo* interactions between DkERF and promoters were measured by dual‐luciferase assay. Error bars indicate SE from five replicates (***P < 0.001).

In order to investigate the possible regulatory linkage between *DkERF* genes and transcription of cell wall‐related genes, the promoters of *DkEGase1*,* Dk*β*‐gal1*,* Dk*β*‐gal4*,* DkPG1*,* DkPE1*,* DkPE2*,* DkXTH9* and *DkXTH10* were isolated (Table S5), using genome walking, due to the lack of persimmon genome information. Analysis of *cis*‐elements indicated that the GCC box and C‐repeat/dehydration‐responsive element (DRE), recognized by *AP2/ERF* transcription factors, were only distributed in some promoters, such as *Dk*β*‐gal4*,* DkPE1* and *DkXTH9* (Figure 6a). Dual‐luciferase assays indicated that DkERF8, DkERF16 and DkERF19 could trans‐activate *DkXTH9* promoters, with 2.13‐, 2.13‐ and 1.74‐fold enhancement, respectively (Figure 6b). However, these *DkERF* genes had limited effects on the promoters of the other seven putative softening‐related genes, although some *in vivo* regulations had the statistical differences.

### DkERF interaction with the *DkXTH9* promoter

**Figure 7:**
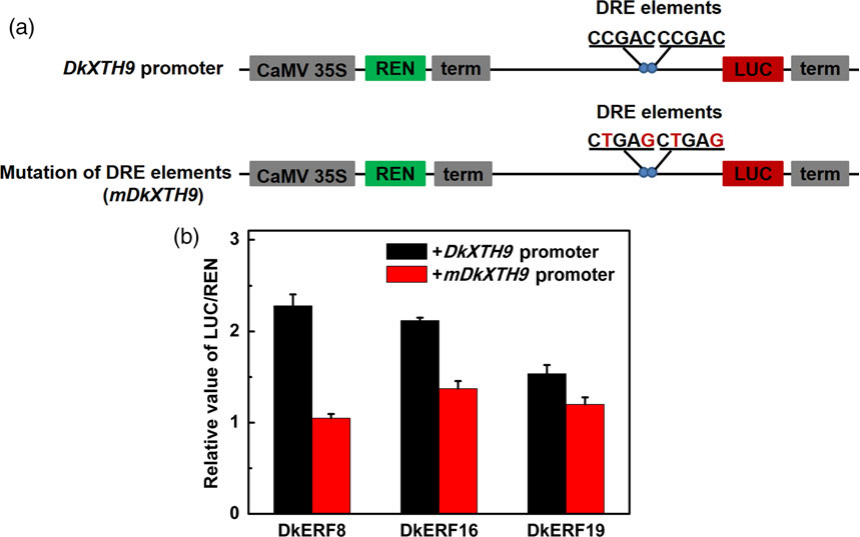
Effect of mutation of DRE elements in the *DkXTH9* promoter on transcription by DkERF. (a) Mutation of DRE elements. (b) Activity of DkERF on transcription from normal and mutated *DkXTH9* promoters. Error bars indicate SE from five replicates.

DkERF trans‐activated the *DkXTH9* promoter, but in a yeast one‐hybrid system, the *DkXTH9* promoter exhibited auto‐activation (Figure S2), so an alternative approach was used to test the interaction. When the two DRE elements (CCGAC) within the *DkXTH9* promoter were mutated to CTGAG (*mDkXTH9*, Figure 7a), dual‐luciferase assay indicated that all three DkERFs had lower trans‐activation activity on the mutated *DkXTH9* promoter, compared to *DkXTH9* promoter (Figure 7b).

## Discussion

### Regulation of anaerobic treatment on persimmon fruit deastringency and softening

For persimmon, postharvest softening not only depends on ripening stage, but also occurs rapidly during astringency removal (Arnal and Del Río, 2004; Nakatsuka *et al*., 2011; Ortiz *et al*., 2005; Taira *et al*., 1992), which is essential because the main persimmon cultivars are of the astringent type (Luo *et al*., 2014; Yamada *et al*., 2002). Thus, although deastringency treatments (including the most widely used high CO_2_ treatment, and also ethylene treatment (Min *et al*., 2012)) remove soluble tannins successfully, they significantly shorten persimmon fruit shelf life. Here, fruit of two Chinese persimmon cultivars, ‘Mopanshi’ and ‘Jingmianshi’, of the astringent type exhibited rapid softening after high CO_2_ (95%) treatment, which is similar to the results from other persimmon cultivars, such as ‘Rojo Brillante’ (Arnal and Del Río, 2004) and ‘Saijo’ (Xu *et al*., 2003). ‘Tonewase’, a Japanese persimmon cultivar, maintained firmness after CO_2_‐driven deastringency (Figure 4). It should note that ‘Tonewase’ obtained from different orchards (Itamura *et al*., 1995) or environments (e.g. humidity, Nakano *et al*., 2002) has been reported to have varied softening rates. The responses of ‘Tonewase’, the Japanese cultivar, to high CO_2_ treatment (same treatment as for the two Chinese cultivars) show that in this cultivar, deastringency and the accompanying softening are effectively uncoupled.

In the Chinese cultivars ‘Mopanshi’ and ‘Jingmianshi’, the softening that accompanies the anoxia‐induced deastringency could be prevented by application of the ethylene antagonist 1‐MCP and softening was retarded while astringency was reduced or removed (Figures 1 and 4), indicating that ethylene signalling was probably responsible for the softening process during deastringency without 1‐MCP. While 1‐MCP had little effect on the removal of astringency, it was extremely effective at preventing fruit softening in the Chinese cultivars, which is to be expected for an ethylene response.

### Characterization of genes involved in persimmon fruit softening during deastringency

Fruit texture is generally considered as a quantitative trait, which is regulated by multiple genes (Li *et al*., 2010), such as *PG* (Atkinson *et al*., 2002; Smith *et al*., 1988), *XTH* (Miedes *et al*., 2010), β*‐Gal* (Kitagawa *et al*., 1995; Nakamura *et al*., 2003; Smith *et al*., 2002) and pectate lyase (Uluisik *et al*., 2016). Here, eight cell wall‐related genes (*Dk*β*‐gal1/4*,* DkEGase1*,* DkPE1/2*,* DkPG1*,* DkXTH9/10*) were identified after a preliminary search and their expression correlated with fruit firmness in CO_2_‐ and CO_2_ + 1‐MCP‐treated ‘Mopanshi’ and ‘Jingmianshi’ fruit and CO_2_‐treated ‘Tonewase’ fruit. The expression of *DkEGase1* in particular was significantly correlated with fruit firmness (Figure 2), indicating the possible involvement of cellulose metabolism during persimmon fruit softening. Furthermore, mRNAs for five other genes (*Dk*β*‐gal1/4*,* DkPE1/2* and *DkPG1*) related to modifications to pectin, one of the main cell wall components, accumulated during softening (Figure 2), which is consistent with previous demonstrations of the large decrease in pectin content during persimmon fruit softening (Cutillas‐Iturralde *et al*., 1993; Luo, 2007; Taira *et al*., 1997). None of these genes, however, has been previously reported in persimmon fruit.

XTH is involved in hemicellulose metabolism and is considered one of the most important enzymes that contribute to persimmon fruit softening (Cutillas‐Iturralde *et al*., 1994). Previously, *DkXTH1* and *DkXTH2* have been shown to be associated with persimmon softening (Nakatsuka *et al*., 2011; Zhu *et al*., 2013), but these two *DkXTH* genes were different genes with same names. Based on the similarity of nucleotide acid sequences, the present *DkXTH1* and *DkXTH2* were similar to *DkXTH1* and *DkXTH2* from ‘Fupingjianshi’ (Zhu *et al*., 2013), while the present *DkXTH8* and *DkXTH9* were similar to *DkXTH1* and *DkXTH2* from ‘Saijo’ (Nakatsuka *et al*., 2011). Here, two *DkXTH* genes (*DkXTH9* and *DkXTH10*) were characterized, which indicated potential overlap between natural postharvest softening and anoxia‐induced softening on *DkXTH9* (which was named as *DkXTH2* in ‘Saijo’, Nakatsuka *et al*., 2011).

### Involvement of hypoxia‐responsive *ERF*s in regulating postdeastringency softening‐related genes

Twenty‐two *DkERF* genes were previously characterized as responsive to high CO_2_ treatment (Figure 3; Min *et al*., 2012, 2014), but only four (*DkERF9/10/19/22*) are transcriptional regulators of persimmon fruit astringency removal by regulating genes encoding PDC and ADH (Min *et al*., 2012, 2014). Thus, only a minority of *DkERF* genes are involved in deastringency, and others, including three *DkERF* genes (*DkERF8/16/19*) were identified as candidates for regulation of postdeastringency softening.

**Figure 8:**
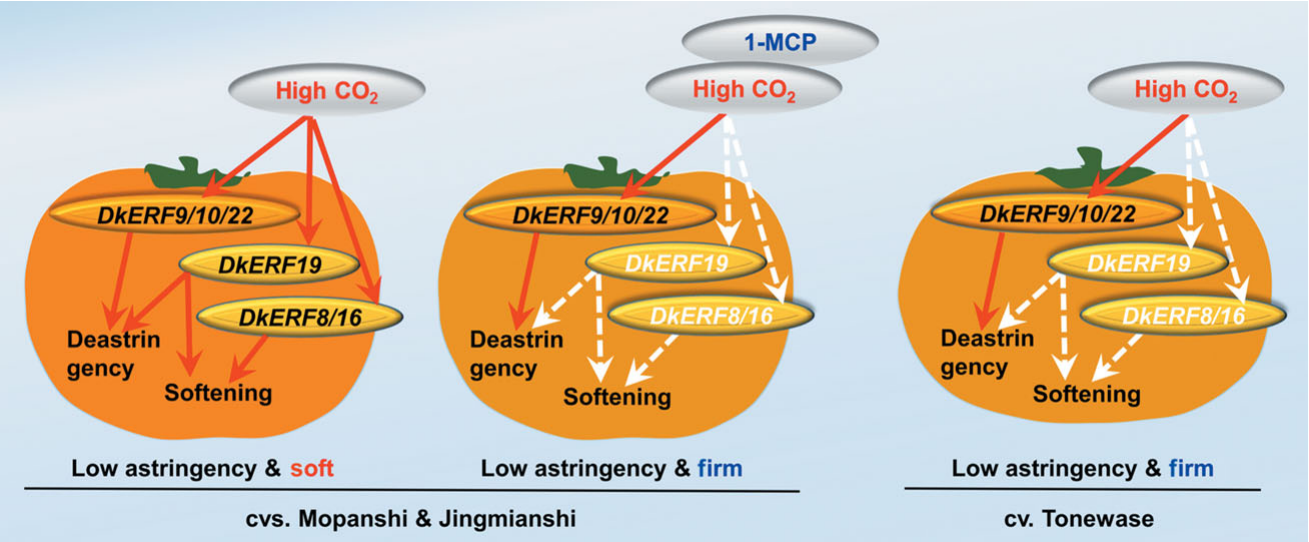
Proposed regulatory model for ethylene response factors in persimmon fruit deastringency and softening. High CO_2_ treatment is an effective treatment for postharvest deastringency and widely used for industry. Six *DkERF* genes were characterized to be responsive to high CO_2_ treatment: DkERF9/10/22 trans‐activate the DkADH/DkPDC and are involved in deastringency; DkERF8/16 are involved in regulation of cell wall metabolism‐related *DkXTH9* and associated with fruit softening; DkERF19 has a dual regulatory role in deastringency and softening. Red arrows indicated significant activations; white arrows indicated the absence of activations.

DkERF8/16/19 were shown to trans‐activate the *DkXTH9* promoter, but not the promoters of the other seven cell wall‐related genes that show increased mRNA accumulation in response to anoxia. In apple, *MdCBF2* can activate the *MdPG* promoter (Tacken *et al*., 2010), and in kiwi fruit, AdERF9 represses the *AdXET5* (Yin *et al*., 2010). In most reported cases, however (including *SlERF.B3*,* ERF2.2*,* MdCBF2* and *AdERF9*), the binding of these transcription factors to target promoters was not addressed. Our results indicate a direct *in vivo* activation by DkERF8/16/19 of the *DkXTH9* promoter, as the effect was greatly reduced by targeted mutations on DRE (a known binding motif for *ERF*) in the *DkXTH9* promoter. Direct binding of transcription factors on targets promoters could be analysed by various methods, such as EMSA. Here, dual‐luciferase assay and motif mutagenesis were also widely used methods to indicate the potential direct binding. Moreover, motif mutagenesis also provided the self‐explanations for *in vivo* effects of DkERF8/16/19 were through the recognization on DRE motif, but not the side effects of genes themselves or infiltrations. These results indicated hypoxia‐responsive *DkERF8/16/19* contributed to the softening that accompanied de‐astringency. Previously, four hypoxia‐responsive ERFs, *DkERF9/10/19/22*, were shown to be regulators of *ADH* and *PDC* and contribute to fruit deastringency (Min *et al*., 2012, 2014). Here, except for *DkERF19*, the other three deastringency‐related *DkERF9/10/22* were also response to deastringency treatment in ‘Tonewase’ persimmon (Figure S3), which were similar to previous findings. A model incorporating these findings is shown in Figure 8. The action of some ERFs could explain the linkage between deastringency and softening, processes which are generally regarded as quite distinct (Figure 8). Furthermore, *DkERF19* has regulatory roles in both deastringency (*DkPDC2*, Min *et al*., 2014) and softening (*DkXTH9*; Figures 6 and 7), and could genuinely be considered to have dual‐functions (Figure 8). Our previous findings (Min *et al*., 2012, 2014) and the present research indicated that *DkERF* genes appear to be involved in both astringency removal and softening of persimmon fruit. The difficulties in generating transgenic persimmon fruit can be circumvented by population screening and other approaches, to identify variants in expression of *DkERF* genes which can be further developed as markers, enabling breeding of persimmon varieties that rapidly lose astringency while maintaining firmness. Meanwhile, ectopic overexpression of the *DkERF* to tomato (a model fruit) is another alternative, but different genetic background might give the different results. The mechanism for this difference in the anaerobic response between the Chinese and Japanese cultivars requires further investigation. Furthermore, although eight cell wall‐related genes were shown to be closely associated with persimmon fruit softening during deastringency, only *DkXTH9* was responsive to the *DkERF* transcription factors and it seems likely that further investigation will reveal other factors involving different signalling pathways, which also participate in softening regulation.

## Experimental procedures

### Plant material and treatments

Three astringent‐type persimmon (*Diospyros kaki*) fruit were selected for this study, including two Chinese cultivars ‘Mopanshi’ (previously named ‘Mopan’, Min *et al*., 2012, 2014) and ‘Jingmianshi’ and one Japanese cultivar ‘Tonewase’.

‘Mopanshi’ persimmon fruit were harvested from a commercial orchard at Fangshan (Beijing, China) in 2012. Fruit without disease or mechanical wounding were selected. Three different treatments were conducted: (i) the first batch of fruit was treated with 95% CO_2_ for 1 day to remove astringency and the post‐treatment fruit exhibited rapid softening, (ii) the second batch was treated with a combination of 95% CO_2_ and 1 μL/L 1‐MCP for 1 day (CO_2_ and 1‐MCP treatments were performed at the same time), which removed the astringency and maintained fruit firmness, and (iii) the third batch of fruit was sealed in containers similar to those in the above treatments for 1 day, as control. The fruit after treatment were transferred to storage in air. All treatments and post‐treatment storage were at 20 °C.

In order to verify the combined effects of 95% CO_2_ and 1 μL/L 1‐MCP treatment (CO_2_ + 1‐MCP), ‘Mopanshi’ and another astringent‐type cultivar (‘Jingmianshi’) were collected from a commercial orchard at Qingdao (Shandong, China) in 2014. Treatments and the conditions were the same as in 2012 and the effect of CO_2_ + 1‐MCP on fruit deastringency and softening was similar to that in 2012 (data not shown).

In the 2014 season, an additional sample collection was conducted with a Japanese astringent‐type cultivar, ‘Tonewase’. ‘Tonewase’ fruit maintain firmness after astringency removal; thus, only CO_2_ treatment and control treatment were performed. The fruit were obtained from a commercial orchard at Qingdao (Shandong, China).

All of the above treatments were designed with three biological replicates (150 fruit in each). At each sampling time, flesh samples (three replicates, three fruit in each) were bulked and frozen in liquid nitrogen and stored at −80 °C until further use (soluble tannin measurements and RNA extraction).

### Fruit firmness

Firmness was measured with a TA‐XT2i texture analyser (Stable Micro Systems, UK), and the penetration indices were calculated according to Yin *et al*. (2012). Over two different seasons, the firmness was measured using two different texture analyser probes, a 7.5‐mm‐diameter probe for ‘Mopanshi’ fruit in 2012 and a 5‐mm‐diameter probe used for ‘Jingmianshi’ and ‘Tonewase’ fruit in 2014. For each fruit, firmness was measured twice at the equator region at 90° intervals, after removal of 1‐mm peel. Fruit firmness was measured with 10 fruit replicates.

### Soluble tannin content

Soluble tannins are the main source of persimmon fruit astringency. Here, measurements of soluble tannin content were made using two different methods. For ‘Mopanshi’ fruit, soluble tannins were measured with the Folin‐Ciocalteu reagent, using frozen samples, according to the method described by Yin *et al*. (2012). The results were calculated using the standard curve of tannin acids equivalents g^−1^ fresh weight. Soluble condensed tannin content was measured with three biological replicates. For ‘Jingmianshi’ and ‘Tonewase’, soluble tannins were visualized by the tannin printing method, according to Min *et al*. (2015). Fruit after treatment were immediately cut lengthwise and then printed onto 5% FeCl_2_‐soaked filter paper for 5 s. After removal of fruit, the soluble tannin contents were observed by the intensity of black colour and the filter paper was photographed.

### RNA extraction and cDNA synthesis

Total RNA extractions were conducted by the methods described by Yin *et al*. (2012). Each extraction was conducted with 2.0 g frozen persimmon fruit flesh. The total RNA was treated with TURBO DNAse (Ambion) to remove the contaminated gDNA, and then used for cDNA synthesis, using an iScript™ cDNA Synthesis Kit (Bio‐Rad). Three biological replicates were used for RNA extraction.

### Gene/promoter isolation

Twenty‐two high CO_2_‐responsive *DkERF* genes were previously isolated using RNA‐seq and RACE technologies (Min *et al*., 2012, 2014; Yin *et al*., 2012). Using the same RNA‐seq data, differentially expressed unigenes potentially related to cell wall degradation were obtained. The full‐length unigenes were obtained, using a SMART RACE cDNA amplification Kit (Clontech). Promoters of cell wall‐related genes were obtained using the Genome Walker Universal Kit (Clontech). Two DRE motifs of *DkXTH9* promoter were mutated using the Fast Mutagenesis System Kit (Transgen). All primers used for gene and promoter isolation are described in Table S1.

### Real‐time PCR analysis

For real‐time PCR, gene‐specific oligonucleotide primers were designed and are described in Table S2. Gene specificity of each pair of primers was double‐checked by melting curve and PCR product re‐sequencing. The *DkACT* was chosen as a housekeeping gene to monitor the abundance of mRNA (Min *et al*., 2014).

Real‐time PCR reactions were performed on a CFX96 instrument (Bio‐Rad). The PCR program comprised an initial step at 95 °C 3 min, 45 cycles of 95 °C 10 s and 60 °C 30 s, ending with a melting curve analysis programme. The PCR mixture (20 μL total volume) consisted of 10 μL Ssofast EvaGreen Supermix (Bio‐Rad), 1 μL of each primer (10 μm), 2 μL of 10‐fold diluted cDNA and 6 μL H_2_O. No‐template controls and melting curve analysis were included for each gene during each run. 2^−ΔΔCt^ method was used to calculate the relative expression levels of genes (Livak and Schmitten, 2001).

The heatmap was used to present the genes expression results and was constructed by MetaboAnalyst 3.0. The different colours indicated up (red) or down (blue) regulations. mRNA abundance was indicated with the intensity of colours. The A, B and C represent three biological replicates.

### Dual‐luciferase assay

Dual‐luciferase assay was used as an efficient and rapid method to detect *in vivo* trans‐activation or trans‐repression effects of transcription factors (Yin *et al*., 2010; Zeng *et al*., 2015). Full‐length *DkERF* genes were fused to pGreen II 0029 62‐SK vector (SK) by Min *et al*. (2012, 2014). Due to the lack of availability of a persimmon genome, promoters of *Dk*β*‐gal1/4*,* DkEGase1*,* DkPE1/2*,* DkPG1* and *DkXTH9*/10 were obtained with GenomeWalker™ Universal Kit (Clontech), using the primers described in Table S3. The promoters were amplified with the primers described in Table S4 and were cloned to pGreen II 0800‐LUC vector (LUC). Details of vector information are described in Hellens *et al*. (2005).

All of the constructs were electroporated into *Agrobacterium tumefaciens* GV3101. The dual‐luciferase assays were performed with *Nicotiana benthamiana* leaves. *Agrobacterium* cultures were prepared with infiltration buffer (10 mm MES, 10 mm MgCl_2_, 150 μm acetosyringone, pH 5.6) to an OD_600_ from 0.7 to 1.0. *Agrobacterium* culture mixtures of transcription factor and promoter (v/v, 10 : 1) were infiltrated into tobacco leaves using needleless syringes. Tobacco plants were grown in a growth chamber, with light : dark cycles of 16 : 8 h. Three days after infiltration, firefly luciferase and renilla luciferase were assayed using the dual‐luciferase assay reagents (Promega). The results were calculated from at least five replicates.

### Statistical analysis

Statistical significance of differences was calculated using Student's *t*‐test or least significant difference (LSD) using DPS7.05 (Zhejiang University, Hangzhou, China). Figures were drawn using Origin 8.0 (Microcal Software Inc., Northampton, MA).

## Conflict of interest

Authors have no conflict of interest to declare.

## Supporting information


**Figure S1** Expression of thirty‐five cell wall‐related genes in response to CO_2_ (95%) and CO_2_+1 ‐ MCP (1 μL L^−1^) treatments in ‘Mopanshi’ persimmon fruit at 20 °C.

**Figure S2** Auto‐activation test for *DkXTH9* promoter.

**Figure S3** Expression of *DkERF9/10/22* genes in response to CO_2_ (95%) treatment in ‘Tonewase’ persimmon fruit at 20 °C.

**Table S1** Sequences of the primers used for gene isolation.

**Table S2** Sequences of the primers used for gene expression analysis.

**Table S3** Sequences of the primers used for genome walking.

**Table S4** Sequences of the primers used for promoter amplification.

**Table S5** Sequences of the promoters of cell wall‐related genes from ‘Mopanshi’ persimmon.
