# Supplementary material for: Hypoxia‐responsive ERFs involved in postdeastringency softening of persimmon fruit
Source: Plant Biotechnol J. 2017 Apr 11;15(11):1409–19. doi: 10.1111/pbi.12725 (PMC5633758; doi:10.1111/pbi.12725)
Supplement: Supplementary file 1 — Figure S1 Expression of thirty‐five cell wall‐related genes in response to CO2 (95%) and CO2+1 ‐ MCP (1 μL L−1) treatments in ‘Mopanshi’ persimmon fruit at 20 °C. [file PBI-15-1409-s006.pdf]

Supplemental Fig. 1

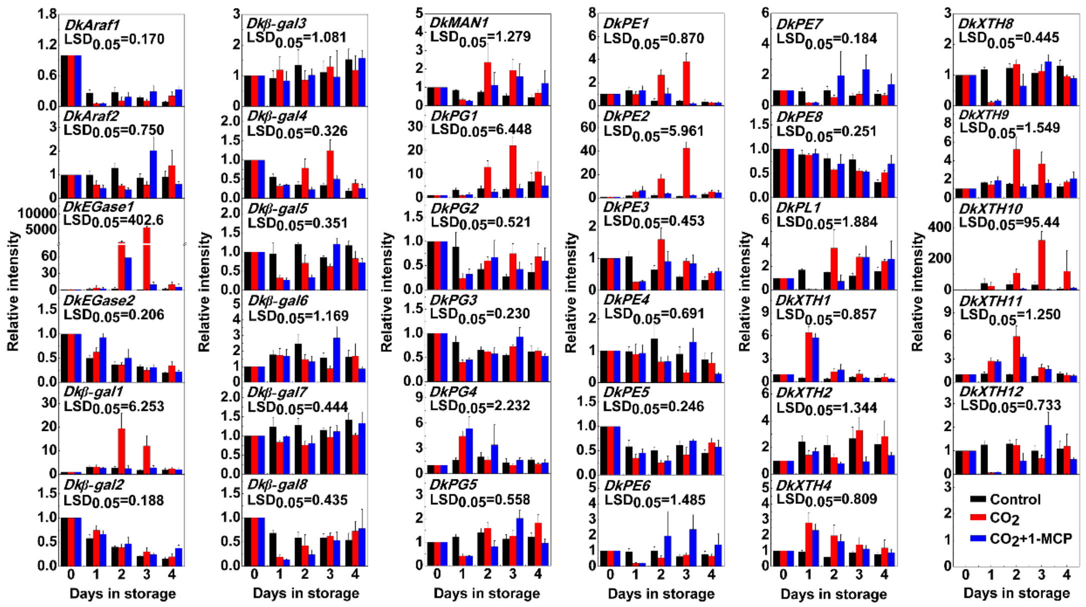

Supplemental Figure 1. Expression of thirty-five cell-wall related genes in response to CO_2(95%) and CO_2+1-MCP (1 μl/L)treatments in 'Mopanshi' persimmon fruit at 20 ^{o}C.
