# Supplementary material for: Hypoxia‐responsive ERFs involved in postdeastringency softening of persimmon fruit
Source: Plant Biotechnol J. 2017 Apr 11;15(11):1409–19. doi: 10.1111/pbi.12725 (PMC5633758; doi:10.1111/pbi.12725)
Supplement: Supplementary file 2 — Figure S2 Auto‐activation test for DkXTH9 promoter. [file PBI-15-1409-s005.pdf]

Supplemental Fig. 2

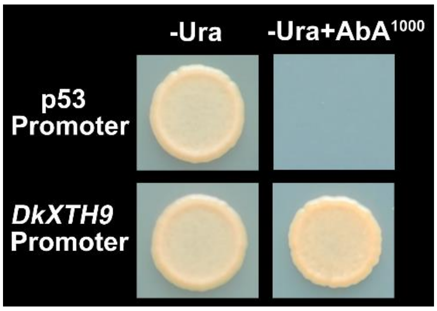

Supplemental Figure 2. Auto-activation test for *DkXTH9* promoter, using the MatchmakerGold Yeast One-Hybrid Library Screening System(Clontech, USA). Auto-activation of promoters were tested on SD medium lacking Ura in presence of aureobasidin A.
