# Supplementary material for: Hypoxia‐responsive ERFs involved in postdeastringency softening of persimmon fruit
Source: Plant Biotechnol J. 2017 Apr 11;15(11):1409–19. doi: 10.1111/pbi.12725 (PMC5633758; doi:10.1111/pbi.12725)
Supplement: Supplementary file 3 — Figure S3 Expression of DkERF9/10/22 genes in response to CO2 (95%) treatment in ‘Tonewase’ persimmon fruit at 20 °C. [file PBI-15-1409-s004.pdf]

Supplemental Fig. 3

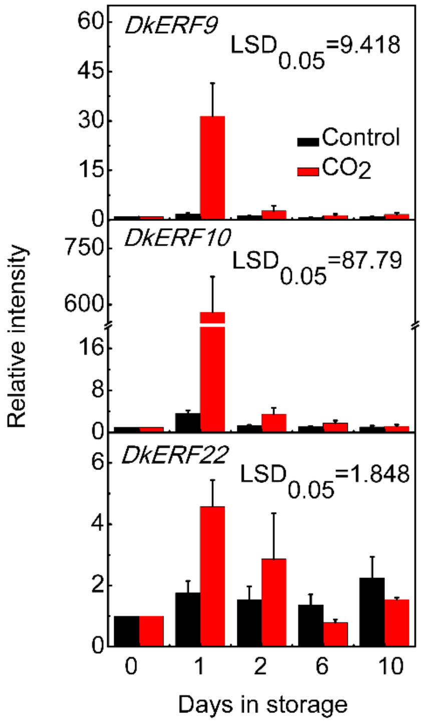

Supplemental Figure 3. Expression of *DkERF9/10/22* genes in response to CO_2(95%) treatment in 'Tonewase' persimmon fruit at 20 ^{o}C. Gene expression was analyzed by realtime PCR. Error bars indicate SEs from 3 replications
