# Supplementary material for: Hypoxia‐responsive ERFs involved in postdeastringency softening of persimmon fruit
Source: Plant Biotechnol J. 2017 Apr 11;15(11):1409–19. doi: 10.1111/pbi.12725 (PMC5633758; doi:10.1111/pbi.12725)
Supplement: Supplementary file 4 — Table S1 Sequences of the primers used for gene isolation. [file PBI-15-1409-s007.pdf]

Supplemental Table 1

Sequences of the primers used for gene isolation

	Gene	Primary PCR (5' to 3')	Secondary PCR (5' to 3')
3'RACE	*DkAraf1*	ATGCAGCCAAGGGACCTGTGATTCT	ATACGCAGAGACGGTCCCAATGACA
	DkEGase1	TGATGCCGGGGATAACGTCAAGTTT	AGGTGAAGAGGCCAAACTGCGTCAT
	DkEGase2	AAATATGGCGACGCCCTGAAAACTG	ACTGCCACCGTATTGTCCTGGGCTA
	Dkβ-gal1	TCTTTTCGCCGTTGGTTGTGATGAA	AGGCAACCAAAATGGGGACATTTGA
	Dkβ-gal2	TCACAGGCCACCTGAAGATGTTGCT	CCAGAAAGGTGGCAGCTTGCAGAAT
	Dkβ-gal3	TTCCTTCTCTCTCTCGTCGCCTTCG	GGGGATTTGCACTATTTCCGGGTTC
	Dkβ-gal4	TACGGTGGCTTTCCTCTTTGGTTGC	AGTACCATGGGTGATGTGCCAGCAA
	Dkβ-gal5	CCACGATCCTGGTTACAGGCATCAA	CAGTGCGATGATGGGCATCAGATTT
	Dkβ-gal7	CATGCTGCTCTTGCTGGTTGGATTC	CAATTGATAGCACACCTGGCCTCCA
	DkPE2	TGCTCATCTCTGCCCCTTTTGGTCT	AACCCCGGGATCTACAAGGAACAGG
	DkPE3	GAGGGACCGAGTATTGCTCCAGACG	GAAAGACGGGACAGGAACGGTCAAG
	DkPE4	ATTTGTGCTTCCCGGCGATTATTCC	GGACGCGCTCAAGAGCAACGATATT
	DkPE6	AGCGGTCTGACCATCCAGAACACAG	CATCTTCGGCAATTCCGCTTCAATC
	DkPE7	TACAGGCCTCCAAGGCCAACTTCAC	GTGGCCAGGTTATCGGGTCATCAAT
	DkPG2-	CCCTACCTTCGTATGGCCGAGGACT	TATTCTCCACCCGAATCGCCCTACA
	DkPG3	GGGATTGAAGTGCCAGGTCGAAGAT	ACTATTGATGGCCAGGGTTCCGTGT
	DkPG4	GGCACAACCCCAACATGTTCTGGT	CAGTAGAGGCGCCAATGATCACCCA
	DkXTH9	CAATGTGTTTACGCGAGGTGGAGGA	ACCCAAGCTCCCTTCACTGCTTCCT
	DkXTH10	CAAAACCGGCTCCATCGCATTTAAC	GGGAACATCATCGGAAGGGAGATGA

	DkXTH11	CGCTGTTGATCCCATCCAAGAGGTT	CTGTTCCACCGCCAGAATGTGTGAT
	DkXTH12	GCCTCAGGTTCGAGTTTCCAGTCCA	CTTGTCAGTGGATGGCACGGTCATT
5'RACE	*DkPE1*	GGCGATGAATCCTTGCCCTTTAACC	GGCAGAAACAGCATCCATCAGCTTG
	DkEGase1	ATGCCAGGAGGAGGAATGTTGAGGA	TGAAGGAGTTGCTGTCGTCCTCTGC
	DkXTH12	CCTCTTGTGGCCCAGAAATCAGCAT	GAATTCGCTTTGGGGTCCAGAGGAT
	DkMAN1	GCTGGCTTTCGTCGCTTTCTCCTGC	ACCGAGGGATCCGAAGTGCATCTGG
	DkPG5	TCAACCACCTTCCCGCCGGCACAAT	ATCGCCGGGTACTCAAAGCATCCCC
	DkPE8	GTACTCCAACGTGTCCAGCGCAAAG	GTGACCATGTTCTTCTGGCCGGAGT
