# Supplementary material for: Hypoxia‐responsive ERFs involved in postdeastringency softening of persimmon fruit
Source: Plant Biotechnol J. 2017 Apr 11;15(11):1409–19. doi: 10.1111/pbi.12725 (PMC5633758; doi:10.1111/pbi.12725)
Supplement: Supplementary file 5 — Table S2 Sequences of the primers used for gene expression analysis. [file PBI-15-1409-s001.pdf]

Supplemental Table 2

Sequences of the primers used for gene expression analysis

	Gene	Primary PCR (5' to 3')	Secondary PCR (5' to 3')
	DkAraf1	GGAGCAGGAAAATTACCTTGG	TGCTCATAACTGTCCGTGAGA
	DkAraf2	TGAAGCGACTGCTAGAATGC	GATTTGCTGCTTCATGACAA
	DkEGase1	TCATGATATTGAACACCTCTGG	CAACACTTCAACAGCCCTACA
	DkEGase2	TGCCATTTCCTTCTTGGAAC	AAGCATAAATGGCTACAAAACG
	Dkβ-gal1	TACCAAACTTACCGGCTTGG	TTAGCCCCCTCTACCTTCGT
	Dkβ-gal2	GCCACCTGAAGATGTTGCTT	TCATCAATAGGCGCATCGTA
	Dkβ-gal3	TTCTCTCTCTCGTCGCCTTC	GAACCCGGAAATAGTGCAAA
	Dkβ-gal4	GATGTGCCAGCAATCAGATG	GCAAAAGCAAGGTCTTCCAC
Real-	*Dkβ-gal5*	ATGGTGTCTCAGGCATGTCA	TGCGTTAGGATGTCATCTGC
time	*Dkβ-gal6*	TGGGAGCATTCATTCAGTTG	CCATAACCACTGATGGGCTA
PCR	*Dkβ-gal7*	AAGATTGCAACGGATCCAAC	TGATCAACCCATTCCCAAAT
	Dkβ-gal8	GGCTAAAATGTGCCCCTGTA	CTATCCGATCACGCTGACAA
	DkMAN1	TTCAGGGACGGGTATGATGT	AACCGTGTTTCGTCAGTTCC
	DkPE1	ATTACATCGATTGTGGGTTTTG	TGCATGCATATTTACCATCAA
	DkPE2	ATCTCCATCGCCTGGCTTT	CTTTCAATAAATGGTGTGAGCA
	DkPE3	CGGTTCATTATCCACGTCAA	CGAAAGATGCAGTGTGGAAA
	DkPE4	TGTATTGATGAGGCACCTACAC	AAAAAGGCAATACATATTTTTCACA
	DkPE5	AAGGGAGGGAGCCTCTGTTA	TGATCAAGAGCCATTGTGGA
	DkPE6	TGCTCACTCTTCTAGCAGTAGGC	AATCTCTAGTTCCATGGGAAAGT

DkPE7	CTGGAATGGAACACCACCTT	CCAACTGGAGCCGAAAATAA
DkPE8	GCTGGACACGTTGGAGTACA	TCCAAGTTCGTACGGAAACC
DkPG1	ACGAAATTCACCAGGGATTG	TTGGTACATACATGGGGGAGT
DkPG2	CTTCCTTGTTCCCATCTCCA	GTTGGTGTAAAGGCAAAACACA
DkPG3	ATTGAAGTGCCAGGTCGAAG	CAACAAGATGAGGACGGCTA
DkPG4	GTTTTGCGGAAGGGGTAACT	ACATAGCCTGCAAGCAAACC
DkPG5	CAGCCAGGTTTGAAGGGATA	TCTGGCAGTAGCTCACAAGG
DkPL1	TCAACTCCGCTTTAGTGGAAA	AGCAAATCCAAGCGACAGAT
DkXTH1	GCTTTCCTGAGGGGCTTC	TCCTTCTCCATCCACAAACG
DkXTH2	CCGAGTGCAAAAGAGACAGA	GCATTGCATCACAACAACATC
DkXTH4	CTCCCCCTGAATGCAGACTA	AACAGAGTTTTCACAGGGGATT
DkXTH8	GCCAGGATTCTCAACAATGG	TTGTATTGAGGAAGGGCATGT
DkXTH9	TGCGTTCTTGTTGTCTCACTTT	GGCTGTGTAAACATGCAGGTAG
DkXTH10	AGAGTTTGGTGAAACATCCAGT	TTGGACGCTCATCTACTACTTTC
DkXTH11	TGAATTTTCTTTGGGTTGCAT	TGTCGTGGCCAAGTAACATT
DkXTH12	TGGTCTTTTGTACTTATCACAATTC	GACTGGAACCTGCCAGTAATTT
