# Supplementary material for: Hypoxia‐responsive ERFs involved in postdeastringency softening of persimmon fruit
Source: Plant Biotechnol J. 2017 Apr 11;15(11):1409–19. doi: 10.1111/pbi.12725 (PMC5633758; doi:10.1111/pbi.12725)
Supplement: Supplementary file 6 — Table S3 Sequences of the primers used for genome walking. [file PBI-15-1409-s002.pdf]

Supplemental Table 3

Sequences of the primers used for genome walking

	Gene	Primary PCR (5' to 3')	Secondary PCR (5' to 3')
Genome walking	*Dkβ-gal1*	CGAACCAGATCATACCTCCCCTCAAAA	TCGTAGGACACAGAGGCTGAGACCAAA
	Dkβ-gal4	TCTGGAGTGCTGCGAGGATAATGAATG	GCGACAAGGCCGACCGTCAAAAGCCAC
	DkEGase1	CCATTGGCAACCCAAACTTGACGTTAT	CGGCCATGCGGATGGCAAATGCACTGC
	DkPE1	ATCGAGCAGATCAAGGCAGTCGAGAAT	GAAGCCGCCATGGAAGAGAAGCGGCGG
	DkPE2	TGTTTCGATTAACTTGGCACGCATCTG	TGAGCAAGAAAGCAACCAGAAATGGTG
	DkPG1	TCATACGCGAATCATAACCAGCTCGAA	GTGGTGCCATTGTATACATATAGAGTTG
	DkXTH9	AGAAGAGAGCCAAAGCGAGAAGCATCA	GAAGACATGGATGGCTGGACTCTGCAA
	DkXTH10	TTTCGAAAGCCTTGGTTAAATGCGATG	ACCATGGTGCGCAGTTGCAGAGGGC
