# Supplementary material for: Hypoxia‐responsive ERFs involved in postdeastringency softening of persimmon fruit
Source: Plant Biotechnol J. 2017 Apr 11;15(11):1409–19. doi: 10.1111/pbi.12725 (PMC5633758; doi:10.1111/pbi.12725)
Supplement: Supplementary file 7 — Table S4 Sequences of the primers used for promoter amplification. [file PBI-15-1409-s003.pdf]

Supplemental Table 4

Sequences of the primers used for promoter amplification

	Gene	Primary PCR (5' to 3')	Secondary PCR (5' to 3')
Promoter amplification	*Dkβ-gal1*	CAGGTCGACTATTAAGTTTTTTTTTATAG	CAGCCATGGGGATGATACAATGGGATGGG
	Dkβ-gal4	CAGTCGACAAATGAGCAACCTGTGACC	CCTCCCATGGTCATGGCGATGAGAAAC
	DkEGase1	GACGTCGACATTAGAAGCTGCATGTAT	GCACCGCGGTTTTTTCTTCTTTTACGAG
	DkPE1	ACGTCGACACCCGCGTTTCGGGCCAAATT	GAAGCCGCCATGGAAGAGAAGCGGCGG
	DkPE2	GATGTCGACTCCAACCTTAGCCTAAGA	TGCTGCCATGGATGGCGCAGCTCAATC
	DkPG1	GAGGTCGACCCTCTAAACTCGTTGTGCC	GCTCCGCGGATAGAGTTGTTTATTTGCT
	DkXTH9	GTGGTCGACAAATCGGCCTGCCTTGAA	GAAGCCATGGATGGCTGGACTCTGCAA
	*DkXTH10**	Same to genome walking	
