# Supplementary material for: Hypoxia‐responsive ERFs involved in postdeastringency softening of persimmon fruit
Source: Plant Biotechnol J. 2017 Apr 11;15(11):1409–19. doi: 10.1111/pbi.12725 (PMC5633758; doi:10.1111/pbi.12725)
Supplement: Supplementary file 8 — Table S5 Sequences of the promoters of cell wall‐related genes from ‘Mopanshi’ persimmon. [file PBI-15-1409-s008.pdf]

Supplemental Table 5

Sequences of the promoters of cell wall related genes from 'Mopanshi' persimmon

Genes	Promoter Sequences (5'-3')
DkEGase1	ATTAGAAGCTGCATGTATACAATGGTATGTACAAGACCAGATATAGCATATGTTATTGGCACTGTTA<br>GTAGATTCTTTTCAAATCCAAGAAGGGAGCATTGGAATGTGATGAAGTGGATCATAAGATATCTCT<br>GAGGAACCAGTCATTTGAAACTTTGTTTTGGGAATGAGAAACCCATTCTAGTTGGCTATACGGATTC<br>TGATATGGATGGAGACATTGACACAAGAAAGTCTACTTCAGGATATTTGATCACTTTCGCAAGAGG<br>AGTAGTGGCTTAGCAATCTAAATTGCAAAAGTGTGTTGCACTTTCTACTACTAAGTCATAATTCAGT<br>GCGATAACTGAAACAGGAAAAGAGTTACTTTGGATGAAGAGATTTATGCAAGAACTTTTTTTTCAA<br>CAAAATAGGTATGTCTTATTTTGAGATAGTCAAAGTGCTATTCATCTTGGTAAAAATCTAACTATTC<br>ATGCTATATCAAAACATATTGATGTAAGGTATCATTGAATACGAGATGTTCTAGATGCTAAGCTGTT<br>AGAACTTGAGAAGGTTCACACTAATAATAATGCTTCTGATATGATGACAATAGCGTTACCAAGAGG<br>GAAGTTTGAGTTATGCTATTCCATTATGGGAGTAGCAAAATCTTCCACATAGTGGAGAGAAGAAGA<br>ATTGTTGGATTTTTTTGGGTCCCTTCCTATGTGGAGAAGCCCAAAAATTATTTGGCCCATGAGCAAGA<br>AGCCCAAGGCCCTTAGGGGTTTCTAAAATTTCAGTTTAAAAGGAGAAAAGGTGTGTGTGTGGGTAG<br>CTATGTGTTTTTGAACGAGAGAAGATAGGAGAGTTGGAAAAGAGGTGTGTGGAATGTGTAGGTTTG<br>ATCAAGTGATCAATTGAGGCATCATTCATGGTTCAATCTTGCTGGGTTTTGGTTAGCTTGTAGAAGA<br>CATCTGGTTCTTCATTTTTAATGGTCAGATTAATGTTTTGAGGTTTTCAACTCCAGTTATTGGTCTCC<br>GAACAAAGGTTTTGTTGTCTGGAATTTTCTAAGTTGAAGAGTAGCGAACAGTTTCTGATCATTTGTA<br>GATTAGAGAGTCATCCATGGTCCGATTGGGCTGATTTTTGAGCTTTACAACTCCACTTTTGTACTCTG<br>TAATAGCAGCCTAATTTCGATGATATATCTCTAGTATCTCTTTTTGTGTTATACTTTGAAGTTGTTCA<br>TGATAGTTAGCCAATTTGAGGTTGTGTACCTGCTGTTGCTCCTGAACAATTACTTGTAGAAGCTTATT<br>ATTCATAGTGGAGTTTTTGGAGTGGACCATTGGGTCCCATGATTTTTACCTTTTCACATTGGAAGGG<br>GTTTTTCACATTAAAAATTGCTTATCTTGTACGTTGAAGTTTGTTCTTTCTTAACTTATTTTTGTACT<br>ACATATATTGTGGTATTTGTTGTGTTATTGTGTTCACTCCTCACAGGTTCAATCAAATAGAGAGAGC<br>TTGTGAATCTTTGGTTACTCTCTCTCTCCCATCAAAAAGTAATGTAATCAAATGCAATAGTAAATGA<br>TGAAAATGGACCCTTTTTATTTGGGAGATTTTTTTATAATATATATAATGGAAAGGGTTTTTAAAAC<br>GTGTGCTTCTAATCTCCCTAAATTGGAGGATATTAGGTGTAAATGAGAACCAGTCTAATCTATTTTA<br>TTTCCTTAATTTCTTTTCCATCCATTTATATTTTATTTCATTCTATTTTATTACTGAATTAATCTATCAC<br>AAACACAGCCTAGATTGCTATAAGGTTTCAGGAAAATGATTTGATATGACAATTGCAGTCGATATC<br>TTCTCATTGTCCAAACATAGATGACACCAAAATGTAGAAAAGGGGAGAGAGAGAGATCGACCAAG<br>ATTGTCATGTGGGGCAAAGAATAAGAACCAGTTGACACACGAGACTAAATAGTAAGGACTGTAGG<br>ATTAATTCCCTGTTCTTGTCATGCCTCACTCTCAGGTTGCTTACTTCAACAAGATCACCACAAGTATG<br>GCCGGTTTGGCACAGTTAATCCCGTGATTGACATAACGTCTTTCTATCTCTCTCTCTCAGCAAACT<br>TGTTTCTCCTCTATGAATACCTCCGCGCCCAATCAAAGCCCAACCCACCACCGCAGCATAAGTGTTC<br>TGAGATTGACAATACAGTCTCGTAAAAGAAGAAAACCATG

Dkβ-gal1	TATTAAGTTTTTTTTTATAGTTAAAATCTAACTTATTTAAAATAAATTTAGCTTAACTAAAAATTAAC<br>TAACGCAATTTGTGATGATAAATAAAAATATAAATCACAAAAGTTTTTAGAGATGTTTTGAAAGCA<br>AAGAAGATTTTTGTGAAAACGGCCGAAACCCAAGATTTAAGACAAATTTATCCTTTAAAGTTATAT<br>ATGTGTTACCTAATATTATCATCTATAAATTATTATGCATATTCACTAAATTTGAAAATATAAAATA<br>GATATATAAAATAAAATACTAGGATTGGAGCAGGCAACAAGTAACTTGGAGAGAGAGAGGGGGAC<br>CCACCTAACAGCAAGGGGACCCACATCCTAAATTTATAAAAGCAGTTGGAGTTGGAGCAGCTTCAC<br>ATTAAAGCCAAACTCAAACCAAACAACTTCCACCCAAAGTTGCCTTCTGTTCTTTTAAACGCGTGTC<br>TTCTGCAACTGCGAGTTTTAACACTTCCTTTTAGTTTTAGTACTACACAATTATCCACGCAAACGCTT<br>TCATTCCCTCTCTTGTTCCTTTCCGGGAAAATAAATAAACGAGGGAATATAAAGTGGACAGAACCT<br>ACGAAGAGAAAAAGGGAAGATTGCCTCGAGCCCATCCCATTGTATCATCCCCATGG
Dkβ-gal4	AAATGAGCAACCTGTGACCCCTAATCCCAAGTCTATTTGTTTTGTTGGGTAGTAGGAGTGGAATCGG<br>TGACTAGTACCTAGATTTGGCGTTGAGTACCTGGAAGCAAAGTTTAAGCTGGGAAATCACCAAATC<br>AAAGAATTTTTAGTTGCTGCTGAATGCTGTCGAGGACTACACGTCAGAAATCACTTTGATGGTGAGC<br>CTCCCGATTATTCGCACCGACTCCATCGGCCTACAACTCCACTACTGCTATGGTGGGCCCAACCAGC<br>TCCCGAGACACGTGGCGATAAAGCACCCTCCTAAATCCATGTGGTCCGTATATTGAAAAAAAATCC<br>TTACTGATCTGCTAACTGAAAGATTATGAAATAATAATTACTTAATGAAGAAATAACAACTGCCATT<br>TGCTACGGTGCCAGCGGTAAAAGTACAGTTGAACCGCGTCGGTAGCGTGAGGGAGATCTGTGGCCA<br>GCGGGGGAAATATACATCAAATGGTGAGGATAAAAATGGGAAAGTGCATTGCAAATGTGAAAGCA<br>ATGGATTGGACGGGCGGAAAATGATCCGTCCCGCAAGAGTGAAGGGAATTGAGTTCTGAGTGAGT<br>GTTGGAAGCGGCAGAGAAAGAAGGACGACGAAGAAGAAGACAGAGCAGAAGCAGACGAAGAGGA<br>TGACACATTCGCGTAAGCATACGAGAGGAGGCGTGTGGTCTCCGGCGTAGGATAGCTTAGCTCCCT<br>TCCTACATTTTGATTCCCTCTCAGTTTCACCTCAGCAATCGTGTTTTCCGTGCGTTTTACAAGGCGGT<br>TAAGCAACGGAAAAGGCATCGCAGCCAAGCAAAGCAAAAATCAAAGTAAATTCTTGCTTCCACGA<br>AAGTGTTTTACGAAAACAGGTTTTAAGAACAGTTGCAACGGCGGAAGGTGTTGTTTCTCATCGCCAT<br>GACCATGG
DkPG1	CCTCTAAACTCGTTGTGCCGGCTCTACCTAAAGTGACCCCATGGGTTGCTCTACTGACATCACTCTA<br>AATCTTTTAACTCTTCAATTGCAATCGACGCAAGGTTATACTTTGAGTCCAACGATCTCAGAATTTT<br>CTCCATGACCTAAACATTGATGATATCTTCTCAATTGATGAACAAGTTCGATTACTCACAAAAAATA<br>ATCGAACATTGATTCTAAGCTTTCTTGGCGCAAAGTTTCAAATTGAGATCTTAAAGTTTGAAAGAGA<br>ATTTTATTTACCTTATCAACTCCACTGAATGTGTTTTGATGAAGCAAATGCCATGCTTCCTTTGATGT<br>TTCTGCTAAGGCAACTAACTCCAACATTGTCTCATCAATTTCTTGGTAAATTGTGTATAGTGCCTTTT<br>TATTCCCTTTTTTTTTTTTTTTTAGAAGCTTTGAGTTCTATCTTTTTTTTGCTTATGACCAACCTACTTTT<br>ACTTTGTTATAATTATGACTCAAAATTTAAATTTGTCTATGGTTGAAATTCTTAATACCGTGATTAGG<br>ATTTTAAATTTAATTGTGATTAGAATGTTGTTGTATTCGATTTTGAGTGTGATTTATTTCATATGAAG<br>AAATATGTTCTTGAATCATTTTTTTATCAGAATATAATTTTTTATGTACATTTATATATTATTTTATGT<br>TTATAAGTAGGTGTCTAATATCTTGAAATTAGTACAACTATATCATTATTATTATACCACTTTTATCA<br>AAGTAATATTCTGTTCTTTTTATCTGTAAGTTTTCTTGAATTGAGTTTTTCACCTAAATTTTTATATTC

	ATTTATGTGATTGATGATTTAATTATAATTTATTTTCTATCCAAATTCATATCATGTGTCATCATCGA<br>TTGGCTCAATATAGCCATTTTGAACAATTTCCCACGCATCTTTTGAACCCAACAGTGCCCTCATTTGT<br>ATGCACCAAATATCATAATTTTGCTTGAAATGAACTTGTGGCCATTTTATATAAAATGACACTCTTA<br>ATTTTCCGTATCAATCACGCGTCAAAGAGCTGCACGCCTGCCTTCTTCTATTTCCTTCTTAATTTCTG<br>CTTCTTCACGTGGGCCCTCCCACTTTTGACCTTTAATTCTCTTTTTATCCAATGCCCACTGCGCACTG<br>TGCACAGACTCCTTCTGGGCTCTGCGAGTTTTCTGGGCTCGACGATCTTCTTGTGTCTTTGACAGCAA<br>CCGGGCTCTGATACCACAATGCTGGAAGATACCCCCTTCCCACTTTCTAAATCAATCACGGTGTTGT<br>GTGAGAGATCAAATGAAGAAGAACAAAGAACTTGTATTTCTTCTTGTATTTCTTCTGATTTTTCATG<br>TTTAGGTACATAACTATTTAATTATGGGCTCAGATTGCAACGGCTCATACAAAAGTCAAATGTCTTT<br>GCTAATTTTTGCTATTAATTTATTCTTCAATTTCTTAACTCTTCAGTTCCAGTTTCTTGTGTCTCTTGA<br>ATTCTCCCACATGCAAGTTTAACATTATTTAACAAGGTGTTCATCTTCATCATCAACTCAAACTTCG<br>ATTATTCACCCTAATGGGTAATTTTAGTTTTCTTGAGTTCAATGTGAATATATATGGGCACAACAAT<br>ATAACAAACATAATTAATTTCGTCTAAATCTCTCCTTTGTCCATACCTCATCGTCACACATGTATAAT<br>ATATAGCATCTACAGATGAATCCCTCCTCTATAAATAGGCAATTGCCATCTTCAAGGGTATTCTATC<br>ATAGATTGCACCAACTGAACCCTTTTTCTAGCAAATAAACAACTCTCCATG
DkPE1	ACCCGCGTTTCGGGCCAAATTCCGCCACTCCTAGCTTTCTAAGGCGGCCGACACGACCCGTTCATAG<br>AGAAAAATAAAGTAGTAACGTCGCTCCCCAATTAATAAGTGATTATGATTTTAGTAAAGTTTTTCT<br>CAGGCACGTAGATAATTTAATATAAGTGGGGCTAATTGTACAAAGTGCAATCTTCAAGAGATCCGT<br>AATTTATTAACCTTTAAAAAGTTCATGCAAAATCACTTTTTTTTTTATATATGATGTTCCCGTTTACG<br>AGCTGCCAGTGCCACCCACAGAGATTCCTACAAACTTTATTTTCTGGGGGCATGAATGACATTTACA<br>TAAATAAAAATTGTATGTAAAAATAGTATAAATTAAAAAAATAAACATAGATAATCCAACTATATA<br>TGGTTATAGAAATGTAATTATTGTTTTATGAATGTAATTTTTTATTAAATATTCATTAATAAGTGAAG<br>TATTAACGCTCCTGCCGCCATTAATGCCCTGCATCTGCCCCCTACCACTTACCCCCCTGACCTCTTTC<br>CATTACCTATTTATGGCAGTCTCCTCTCCACCCTCTCACTCTAATTCTCCATGCACAGTCAATAACAA<br>GCTCTCTCTCTCTCTCTCTCTCTCTCTCTCTCTACAGTCTGCTCTGCCGCCGCTTCTCTTCCATG
DkPE2	TCCAACCTTAGCCTAAGACAACTGTTAATGATCTGTTGCTGTCATCATATGATCACCAACAGTATGG<br>CGATGTAACTAACCATGAATAGTACCCAGAATTAATATTTTGACAAATAATTAACTGGTAGCTAATT<br>AATCTAGATGTGCCCAAGGATCAGGGATTACAAACAAAATCAGCAGTTGTGCGTGCTGCATGGGGC<br>GTTAAGCTTAGACGACGGGGAAGAAAATTGTATTGAGATCAGCTTTCCCAATAAAGAAGATATAAA<br>AATCAATAGCCGGCAACATGTAATACGAACATTGATTTGATGGAATATGCATATATATATGAACTT<br>GAAATTTGGGATTCATAGAACTCATATATATTTACTAAGACATAACTTAAATGGAATTCATTTTCTA<br>TTTTTGATAAATGTGATTTAGTTTATCTTCAACTCTATAGAATTAAATTCTTAGAATTGAAATCCACG<br>CTTACTTTTTTTCATTCCAAATATGCAAATTTGGAATTGGAATACCAAAGCGGAGTGGAAGTTTGGTC<br>CAAAGCATTCAACCCACGGCACAAGCTGAAGATCATCAGATTATTTGGGTATAAAAGAAGTCAATC<br>CGGGTTGTGATTGAGCTGCGCCATCCATG
DkXTH9	AAATCGGCCTGCCTTGAACACGGTAACCTCTATCACTTAATTATAACCATCACATACCGTCCTGAAC<br>CATAAAATAAACAAATAATATGACAGCTTGTATTTGTGATTACAAACGCTTCAATAGGCATCACTCT

	GATAATCTTTTTAAGTAATACCAAGGCACAGCGATCCAATCCTCAGTTGATACACCCATTCCCATGC<br>CACATTTTTTGATTTGGGACTAGAAAACAAAATGATGGCTCCCTCATTTGCTTAATTGACGGCGTGA<br>TTGGAGAATCTCGCGCCGCACGCAAAATAAAACCGAAACCCCACCCCCCCTCCTCCCTCTGCTGTTA<br>TTTATTTAATTATTAAATGTTAAAATTTATTAAACCTTAACTGAGTGAATTTGATTTGAGTTTTGAAA<br>CCCAAAGTTAAATCTTAATAATTTTTATTTTTAATACATAATATTTATTAAATATAACACTTAAATTT<br>AACCAAAAAAATCCACGAATTCCCCAATTATCCGACCCGACGACTGGGGTCCGAAACTTTTTCCAA<br>TTTTGAAGTCAAGCATCCGTTTTTGTTACGCTATGGGTAGTAACGTGTCCCAAGGCAGGTCAATAAC<br>GGTGTCCGGGTCCAAATAAACTCTAAAAAACCCAATATTCAATCCAATCGCTCCTGTTCACACCCAA<br>CTCAAAACCCCCCATTGGAATCCTCCCTCGTTATCCGTGTCCCTCTCTCTTTAACCCATCTCCCCTTC<br>TTCCACCCCCCCCTTCCCCCCCGTTGTTACCCACTTGCAGAGTCCAGCCATCCATG
DkXTH10	GGCCCGGGCTGGTATTATAAAACCTAACATCATATTCAAAACAAAAATAGAACACAATAAACTCAC<br>TCTAAACTAATTTAATAAAACCAAATTTTATTGAAATGGATTCGTGAATATTACTTTTATTCTTTTAA<br>TTACTTGATTAACAAAAAAAAAATGAAAATTGCTTCCCAAATCATCCATACATTGGAAAAGAATCAT<br>CGAAATAGATCATTCAAAATTTTAGAAAATACTATTCCAATGGCATTATAATTTTATCTACAATATA<br>TAAAAGAAAGTCATTTTCCAATTTTCTTGGGTGGGACCTCCTCATCTCCCCTATTTATACAACTGCA<br>ATGCTACACTGCATGCACAGACAGACCTGTGTTCGTTTCCACTCATTTAATTCTTTGGCATTCTTCAT<br>TTAATATTCATCTGCTTTAATTCCGCAGCAGTTTCTTTCTCCTCCCTCTGCCCTCTGCAACTGCGCAC<br>CATG
